# Bioproduction of propionic acid using levulinic acid by engineered *Pseudomonas putida*

**DOI:** 10.3389/fbioe.2022.939248

**Published:** 2022-08-10

**Authors:** Rameshwar Tiwari, Chandran Sathesh-Prabu, Sung Kuk Lee

**Affiliations:** School of Energy and Chemical Engineering, Ulsan National Institute of Science and Technology (UNIST), Ulsan, South Korea

**Keywords:** levulinic acid, propionic acid, *Pseudomonas putida*, glycerol, metabolic engineering

## Abstract

The present study elaborates on the propionic acid (PA) production by the well-known microbial cell factory *Pseudomonas putida* EM42 and its capacity to utilize biomass-derived levulinic acid (LA). Primarily, the *P. putida* EM42 strain was engineered to produce PA by deleting the methylcitrate synthase (PrpC) and propionyl-CoA synthase (PrpE) genes. Subsequently, a LA-inducible expression system was employed to express *yciA* (encoding thioesterase) from *Haemophilus influenzae* and *ygfH* (encoding propionyl-CoA: succinate CoA transferase) from *Escherichia coli* to improve the PA production by up to 10-fold under flask scale cultivation. The engineered *P. putida* EM42:ΔCE:*yciA:ygfH* was used to optimize the bioprocess to further improve the PA production titer. Moreover, the fed-batch fermentation performed under optimized conditions in a 5 L bioreactor resulted in the titer, productivity, and molar yield for PA production of 26.8 g/L, 0.3 g/L/h, and 83%, respectively. This study, thus, successfully explored the LA catabolic pathway of *P. putida* as an alternative route for the sustainable and industrial production of PA from LA.

## Highlights

• *P. putida* was engineered to produce propionic acid from levulinic acid.
• A substrate-based inducible system was used to express heterologous genes to avoid using chemical inducers.
• Carbon flux to propionic acid was promoted by expressing *ygfH*.
• Engineered *P. putida* produced 26.8 g/L propionic acid with 83% molar yield in a 5 L fed-batch bioreactor.

## Introduction

Propionic acid (PA) is a C3 carboxylic acid and a promising second-tier group of building block candidates recommended by the U.S. Department of Energy (Werpy & Petersen, 2004). However, the petrochemical-based process for PA production requires the application of high temperatures and pressures along with toxic chemicals such as ethylene, carbon monoxide, and metal catalysts. This makes the whole process non-renewable and unsustainable, generating many environmental pollutants (Liu et al., 2020). Hence, the conversion of biomass-based substrates into PA by microbial cell factories has emerged as an alternative eco-friendly strategy (Eş et al., 2017). However, biorefinery-based bioprocessing has technical limitations related to the bioconversion process and the efficiency of potential pathways in the selected microbial candidates for large-scale biomanufacturing of PA (Ammar & Philippidis, 2021).

To date, high-level PA production has been achieved through the Wood–Werkman cycle of several candidates belonging to the genus *Propionibacteria* and the reductive acrylate pathway of *Clostridium propionicum* under anaerobic conditions using glucose or glycerol as the carbon source (Gonzalez-Garcia et al., 2017a; Collograi et al., 2022). However, *Propionibacteria* or *Clostridium*-based PA production has several limitations, including slow growth rates and costly downstream processing due to the high amount of by-products generated during fermentation (Ranaei et al., 2020). A co-culture approach was also performed for PA production. In this approach, *Lactobacillus zeae* convert glucose to lactate which is the substrate of *Veillonella criceti*, producing PA with a high productivity rate (Dietz et al., 2013). In addition to these conventional microbial candidates, few attempts have been made to engineer microbial cell factories such as *Escherichia coli* to produce PA (Akawi et al., 2015; Gonzalez-Garcia et al., 2017a; Gonzalez-Garcia et al., 2017b). Mostly in *E. coli*, engineering the native sleeping beauty mutase (Sbm) operon or expression of a heterogeneous Wood–Werkman pathway from *Propionibacterium* has resulted in higher PA production under anaerobic conditions (Srirangan et al., 2013; Gonzalez-Garcia et al., 2020). However, this manufacturing process is adversely affected by the low titer and high by-product generation (Ammar & Philippidis, 2021). Moreover, expensive nitrogen flushing is required to maintain the anaerobic conditions throughout the fermentation process, making the complete process economically unviable (Liu et al., 2016; Li et al., 2017). Recently, L-threonine catabolism was engineered in *Pseudomonas putida* to produce PA under aerobic conditions (Ma et al., 2020; Mu et al., 2021). This pathway could thus be explored for the production of PA under aerobic conditions. Moreover, a synthetic propionate pathway has been constructed in *Saccharomyces cerevisiae* by engineering the L-threonine catabolism, thereby resulting in a strain capable of producing the PA from glucose. However, the resultant PA titer (1.05 g/L) was determined to be quite low compared to those reported in other studies (Kidd et al., 2021). L-threonine is the third-most sold amino acid used in livestock feed, pharmaceuticals, and cosmetics (Liu et al., 2019). As L-threonine is mainly produced via fermentative bioprocesses, it cannot be considered the best substrate to produce PA. Although, this cost issue was tried to address by adopting a sequential fermentation−biotransformation process to produce PA directly from the fermentation broth containing unpurified L-threonine (Mu et al., 2021). In a PA biorefinery, the cost of raw materials is approximately 50% of the total cost (Dishisha et al., 2013). Hence, there is a genuine need to select a low-cost and sustainable substrate for PA production. Levulinic acid (LA) is a γ-keto acid (C5) platform chemical that can be obtained by the acid-catalyzed dehydration and hydrolysis of sugars obtained from lignocellulosic biomass substrates (Isoni et al., 2018). As LA can be produced without the use of expensive hydrolytic enzymes, it decreases the total cost of bioprocesses based on lignocellulosic biomass (Habe et al., 2020). The market price of biologically produced PA is 2.00–3.00 $/kg (Ammar & Philippidis, 2021). On the other hand, using biomass is considered a potential way to decrease the LA price by less than 1 $/kg, through Biofine (Hayes et al., 2006) and other processes (Kang et al., 2018; Meramo Hurtado et al., 2021). Thus, LA can serve as a sustainable product that can revitalize the cellulosic biomass-based biorefinery industry. Furthermore, the production cost of LA from biomass has been reduced by the development of LA production technology (Isoni et al., 2018). Complex sugars obtained from the lignocellulosic biomass are used for microbial fermentation, and their production efficiency decreases through long-term process operations owing to sequential sugar application. However, the use of the LA metabolic pathway offers several advantages, such as the lack of complex metabolic regulations and the production of central metabolic intermediates (Kim et al., 2019). Notably, the metabolic pathway of LA utilization was discovered in *P. putida* KT2440 and further employed in the LA-based biorefineries to produce biologically diverse chemicals (Rand et al., 2017; Sathesh-Prabu & Lee, 2019; Cha et al., 2020).

In the present study, the LA metabolic capacity of *P. putida* EM42 to produce PA was explored. Moreover, an engineered strain of the same was developed by co-expressing heterologous thioesterase (YciA) and propionyl-CoA: succinate-CoA transferase (YgfH) under a substrate (LA)-based inducible promoter system to avoid the requirement of other chemical inducers throughout the bioprocess. Furthermore, the assessment of titer and PA productivity along with a large-scale fermentation process was performed to confirm the efficiency of the engineered strain for LA-based PA production.

## Materials and methods

### Bacterial strains and plasmids

The bacterial strains and plasmids used in this study are listed in Table 1. *E. coli* DH10B was used as the cloning host for all the experiments. *P. putida* EM42 strain obtained from Centro Nacional de Biotecnología (CNB-CSIC, Spain) was used as the parental strain for the gene deletion and heterologous gene expression required for PA production. The genomic DNA of *Haemophilus influenzae* (DSM 11121) was purchased from the German Collection of Microorganisms and Cell Cultures GmbH (DSMZ, Germany).

**TABLE 1:** Strains and plasmids used in this invention.

Strain and plasmids	Description	Source
*E. coli* DH10B	Cloning host	Lab stock
*P*. *putida* EM42	Derivative strain from wild type *P. putida* KT2440	Martínez-García et al. (2014)
EM42:ΔC	EM42 with deleted *prpC*	This study
EM42:ΔE	EM42 with deleted *prpE*	This study
EM42:ΔCE	EM42 with deleted *prpC* and *prpE*	This study
EM42:ΔCE:*yciA*	EM42:Δ*prpC*:Δ*prpE* harboring pPROBE_ LvaR/P_*lvaA*__ *yciA*	This study
EM42:ΔCE:*ygfH*	EM42:Δ*prpC*:Δ*prpE* harboring pPROBE_LvaR/P_*lvaA*__*ygfH*	This study
EM42:ΔCE:*yciA*:*ygfH*	EM42:Δ*prpC*:Δ*prpE* harboring pPROBE_LvaR/P_*lvaA*__*yciA*_*ygfH*	This study
pPROBE_ LvaR/P_*lvaA*__e*gfp* ^+^	pBBR1-ori, carrying *egfp* ^+^ under the control of LvaR/P_*lvaA*_ (levulinic acid inducible promoter) from *P. putida* KT2440, Km^R^	Sathesh-Prabu et al. (2021)
pPROBE_ LvaR/P_*lvaA*__ *yciA*	pPROBE_LvaR/P_*lvaA*_ with Δ*egfp* ^+^:*yciA* from *Haemophilus influenzae* DSM 11121	This study
pPROBE_ LvaR/P_*lvaA*__*ygfH*	pPROBE_LvaR/P_*lvaA*_ with Δ*egfp* ^+^:*ygfH* from *E. coli* DH10B	This study
pPROBE_ LvaR/P_*lvaA*__ *yciA*_*ygfH*	pPROBE_LvaR/P_*lvaA*_ with Δ *egfp* ^+^ *egfp* ^+^:*yciA*:*ygfH*	This study
pQSAK	ColE1-*ori*, *sacB*, Km^R^ and Amp^R^	Zhou et al. (2014)
pQSAK-*prpC*	used to delete *prpC* in EM42	This study
pQSAK-*prpE*	used to delete *prpE* in EM42	This study

Restriction enzymes (Thermo Scientific, United States), Q5 High-Fidelity DNA Polymerase (New England Biolabs, United States), and e-Taq Polymerase (SolGent, South Korea) were used for the cloning and plasmid construction. Electro-competent cells were prepared as described previously (Luo et al., 2016). The suicide plasmid pQSAK was used to construct pQSAK-*prpC* and pQSAK-*prpE*, which were used for the chromosomal in-frame gene deletion based on the *sacB*-negative counter-selection system (Zhou et al., 2014). To delete the *prpC* and *prpE*, the pQSAK plasmid was constructed using the Gibson assembly cloning method, containing approximately 500 bp upstream and downstream homologous regions of each gene. The plasmid construct was then transformed into *P. putida* EM42. After double homologous recombination, the colonies were picked from sucrose-kanamycin plates for negative selection using SacB. The deletion was confirmed by PCR followed by DNA sequencing.

In a previous study, an LA-inducible expression system was developed using the transcriptional activator LvaR and its cognate *lvaA* promoter from the *lva* operon of *P. putida* KT2440. Subsequently, the plasmid pPROBE_LvaR/P_*lvaA*__*egfp* ^+^ was constructed, and LA-based induction was analyzed by expressing the green fluorescent protein as a reporter protein (Sathesh-Prabu et al., 2021). To construct the pPROBE_LvaR/P_*lvaA*__*yciA* and pPROBE_LvaR/P_*lvaA*__*ygfH* plasmids, the *yciA* and *ygfH* genes were amplified from *H*. *influenzae* and *E. coli*, respectively, using the primers described in Supplementary Table S1. The amplified gene products were then cloned by digesting the pPROBE_LvaR/P_*lvaA*__e*gfp* ^+^ with NdeI/HindIII and replacing the *egfp* ^+^ with each gene using the Gibson assembly cloning method. The pPROBE_LvaR/P_*lvaA*__*yciA*_*ygfH* plasmid was constructed by employing a transcriptional fusion of the two genes separated by the RBS sequence. The constructed plasmids were then transformed into the EM42:ΔCE strain to yield the EM42:ΔCE:*yciA*, EM42:ΔCE:*ygfH*, and EM42:ΔCE:*yciA*:*ygfH* strains. All the constructed plasmids were confirmed by sequencing (Macrogen, South Korea).

### Media and cultivation conditions

The LA (Sigma-Aldrich) was neutralized to pH 7.0 by 10 N NaOH and sterilized by autoclaving before use. The Luria-Bertani (LB) medium (5 g/L yeast extract, 10 g/L peptone, and 10 g/L NaCl) and Terrific Broth (TB) medium (12 g/L tryptone, 24 g/L yeast extract; 9.4 g/L dibasic potassium phosphate, and 2.2 g/L monobasic potassium phosphate) were used to cultivate the *E. coli* and *P. putida* strains at 37 and 30°C, respectively, under shaking conditions at 200 rpm. To maintain the plasmid construct, the medium was supplemented with 50 μg/ml kanamycin (Km).

For the cultivation, the cells were streaked on LB agar plates (with or without Km, as required) and incubated overnight under the prescribed growth conditions. Subsequently, the colonies were inoculated into a 5 ml LB medium and incubated for 16 h under shaking conditions. The starter culture (0.5 ml) was then inoculated in a 250 ml flask containing 20 ml of TB medium containing either LA, glycerol, or both, as per the experimental conditions. Glycerol was selected as the co-substrate with LA for the production of PA. Samples were collected periodically for further analysis. All the experiments were performed in triplicate. The conversion of LA to PA was calculated on a molar basis and represented as molar yield (%), where only consumed LA was used to calculate the molar yield to avoid other unknown metabolites involved in the PA production.

For the fed-batch fermentation, experiments were performed in a 5 L bioreactor (MARADO-PDA; CNS, Daejeon, South Korea) with an initial working volume of 1800 ml. The previously described TB medium supplemented with 50 μg/ml Km, was used. The cells were initially grown rapidly on glycerol with supplementation of LA (3 g/L) for induction, and then LA was added after 12 h to initiate PA production. The temperature and agitation speed were set to 30°C and 700 rpm, respectively. In addition, the dissolved oxygen (DO) was maintained by flowing 2 vvm of air. The pH was maintained at 7.0 with 2 M NH_4_OH and 4 M H_3_PO_4_. The LA level was maintained between 5 g/L and 15 g/L by intermittent feeding with 50% LA. Samples were withdrawn periodically to determine the cell growth and concentrations of glycerol, LA, and PA.

### Analytical methods

The growth (OD_600_) of the bacterial strains was observed using a spectrophotometer (Libra S22; Biochrom, UK). For the analysis of glycerol, LA, and PA, the collected samples were diluted, and 20 µl aliquots were injected into an HPX-87H column (Bio-Rad) at 0.5 ml/min and a column temperature of 35°C. The analysis was performed on a Shimadzu HPLC station equipped with a refractive index detector (Shimadzu) and SIL-20A auto-sampler (Shimadzu).

## Results and discussion

### Construction of engineered strain for the production of propionic acid from levulinic acid

**FIGURE 1:**
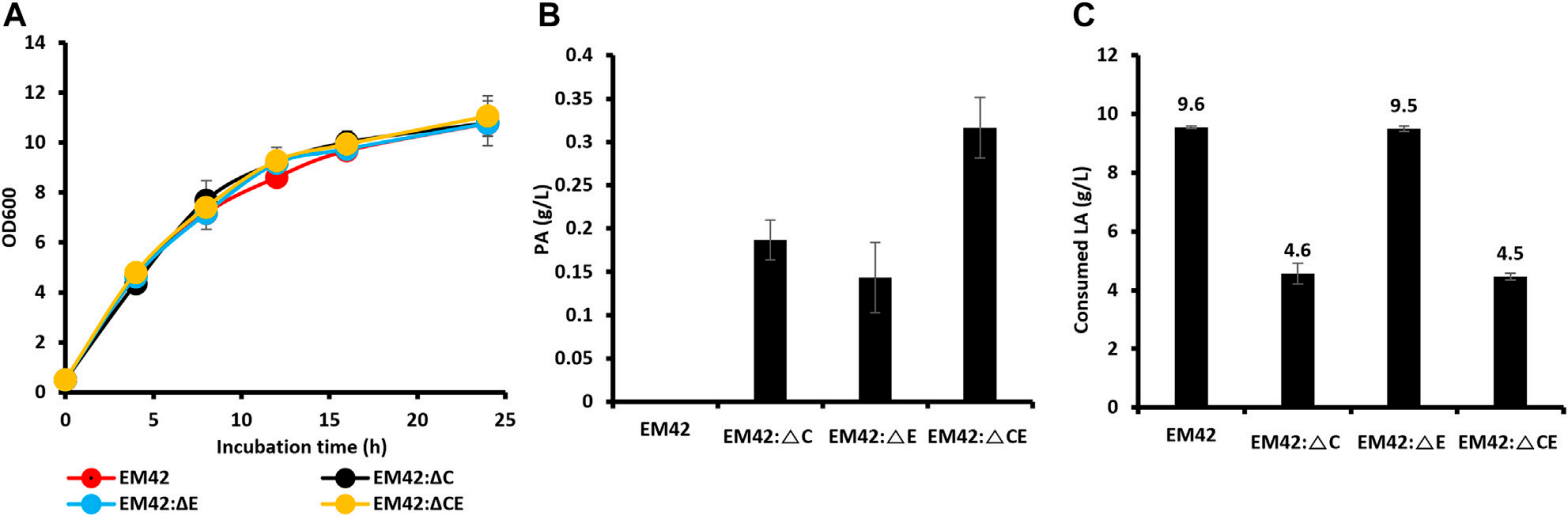
The growth pattern **(A)**, PA production **(B)**, and concentration of residual LA **(C)** by wild type (EM42) and other strains with deleted *prpC* and *prpE*. The presented value is the mean value of three experiments. The standard deviation is represented by error bars.

The *P. putida* EM42 was derived from the platform strain *P. putida* KT2440 by deleting 300 genes (approximately 4.3% of the genome), resulting in increased ATP levels, oxidative stress, growth rates, and enhanced expression of heterologous genes (Martínez-García et al., 2014). As a result, the EM42 strain is more suitable for expressing heterologous genes and producing biochemicals than the KT2440 (Dvořák & de Lorenzo, 2018). *P. putida* strain, as it can utilize LA as the sole carbon source with the aid of proteins encoded by polycistronic genes, designated as *lvaABCDEFG* (Rand et al., 2017). The *lva* operon is upregulated by the transcriptional activator LvaR, which is induced by LA. In this assimilation pathway, LA is first activated by LvaE and a coenzyme A (CoA) thioester, levulinyl-CoA. Subsequently, the LvaD catalyzes the reduction of LA-CoA with either NADH or NADPH to yield 4-hydroxyvaleryl-CoA. The 4-hydroxyvaleryl-CoA is then phosphorylated to yield 4-phosphovaleryl-CoA by the combined action of LvaA, LvaB, and ATP. This is followed by converting 4-phosphovaleryl-CoA into 3-hydroxyvaleryl-CoA by LvaC, which is further oxidized through β-oxidation to yield acetyl-CoA and propionyl-CoA and is completely oxidized through the TCA cycle. Propionyl-CoA can be used as a precursor for PA production, and acetyl-CoA can be directed to the TCA cycle to promote cell growth. Propionyl-CoA can be consumed through the methylcitrate cycle by methylcitrate synthase (PP2335, *prpC*). Moreover, PA can be degraded to propionyl-CoA by propionyl-CoA synthase (PP2351, *prpE*). High production of PA requires an accumulation of its precursor, propionyl-CoA. As a result, *prpC* and *prpE* were deleted from the EM42 strain individually and combined to obtain the EM42:ΔC, EM42:ΔE, and EM42:ΔCE strains. Our growth curve studies demonstrated that these deletions did not affect the cell viability in the TB medium with LA (10 g/L) as the carbon source (Figure 1A). Furthermore, the assessment of PA production after 48 h revealed that while the EM42 strain cannot produce PA, the EM42:ΔCE strain exhibited maximum PA production of 0.31 g/L, accounting for a 9.6% molar yield. Our results also showed that the deletion of both genes allows the accumulation of propionyl-CoA and restricts the degradation of PA to propionyl-CoA, thereby achieving the highest PA among all strains (Figure 1B). However, the LA consumption was affected by these deletions as 48 h post-incubation, the EM42 consumed 9.6 g/L of LA, whereas the EM42:ΔC and EM42:ΔCE strains showed the consumption of only 4.6 and 4.5 g/L of LA, respectively (Figure 1C). This result, thus, showed that the deletion of the PrpC gene instigated the accumulation of propionyl-CoA by blocking the methylcitrate cycle, resulting in lower LA consumption.

### Heterologous expression for improved propionic acid production

**FIGURE 2:**
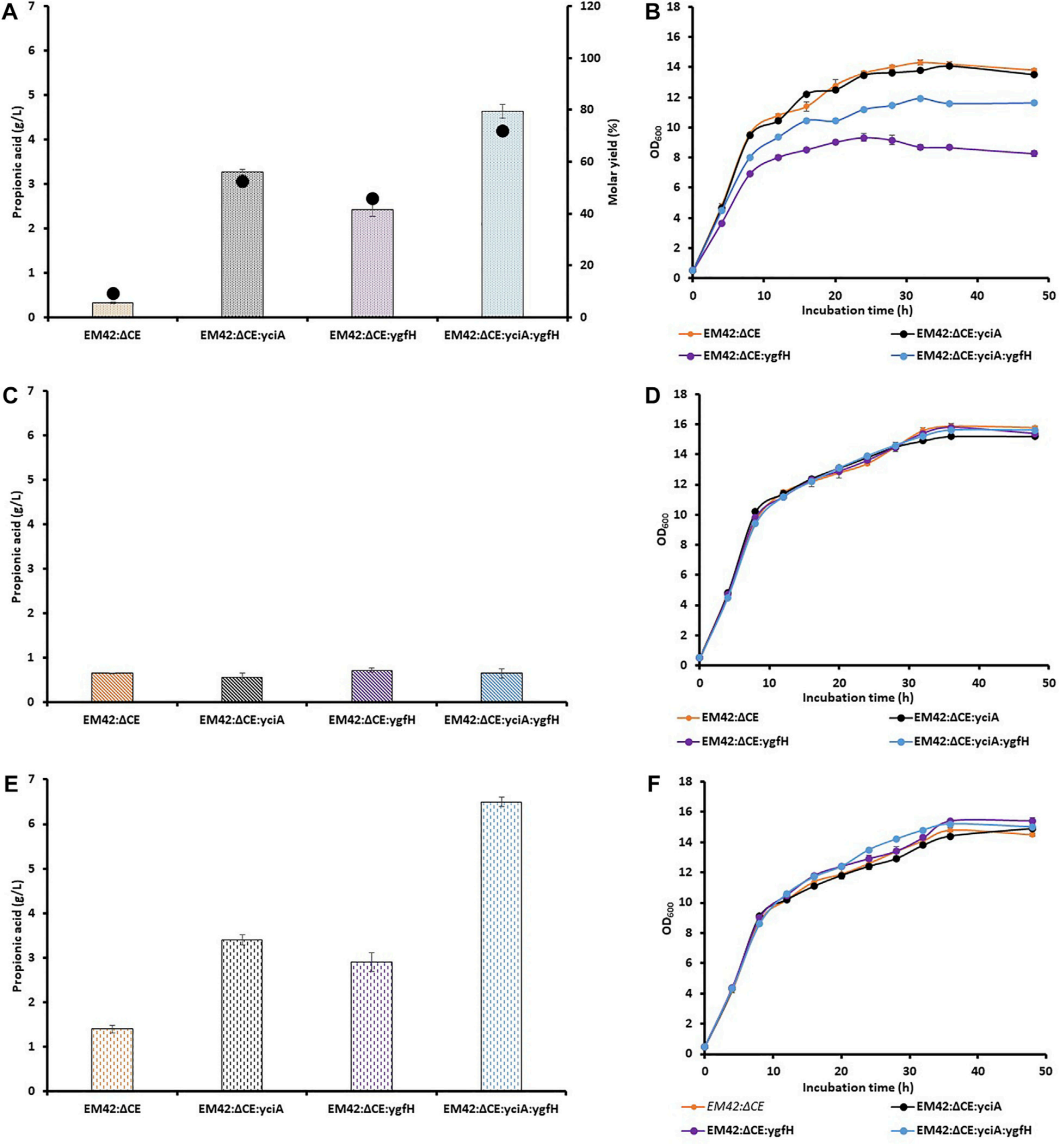
Production of PA and molar yield (%) **(A,C,E)**, and growth pattern **(B,D,F)** of selected strains, including EM42:ΔCE, EM42:ΔCE:*yciA*, EM42:ΔCE:*ygfH*, and EM42:ΔCE:*yciA*:*ygfH* growing on LA **(A,B)**, Glycerol **(C,D)** and LA + Glycerol **(E,F)**. The presented value is the mean from three experiments. The standard deviation is represented by error bars.

Assessment of PA levels revealed that the knockout strain EM42:ΔCE showed significant production of PA as compared to that of the wild-type EM42; however, the production level was quite low. Hence, the conversion of propionyl-CoA to PA can be a limiting step catalyzed by thioesterases. To address this limitation, the well-known thioesterase (YciA) gene from *H*. *influenzae* DSM 11121 (Zhuang et al., 2008) was expressed in an LA-based inducible system (LvaR/P_*lvaA*_) from *P. putida*. This substrate-based induction can avoid the use of costly chemical inducers and the extra metabolic burden on the cell (Ko et al., 2020). Moreover, this promoter system has already been used for the enhanced production of 4-hydroxyvalerate from LA in *P. putida* KT2440 (Sathesh-Prabu & Lee, 2019). The resultant EM42:ΔCE:*yciA* strain utilized LA almost completely (9.8 g/L) after 48 h, which was similar to wild type EM42 and 46% higher than that noted with EM42:ΔCE. Additionally, the expression of exogenous thioesterase also improved PA production from LA by 10-fold compared to that demonstrated by the EM42:ΔCE strain, with a molar yield of 52% (Figure 2A). This result thus, confirmed that the low expression level of indigenous thioesterases was responsible for the lower PA production. Recently, a thioesterase from *P. putida* KT2440, encoded by *PP4975*, was involved in PA production (Ma et al., 2021). However, the activity of thioesterases from *P. putida* KT2440 was quite low in EM42:ΔCE:*yciA* strain.

Even after the successful expression of YciA and enhanced PA production, there is still scope to improve the titer and molar yield. In the engineered EM42:ΔCE:*yciA* strain, the propionyl-CoA was not linked to the central metabolic pathway but only led to its conversion into PA. In contrast, acetyl-CoA is available for the production of cell biomass and other metabolites. In a previous report, 55.5% of LA was presumably metabolized to CO_2_, cell mass, and other products in *P. putida* (Gorenflo et al., 2001). It is not adequate to achieve a high LA-to-PA conversion rate. Overexpression of propionyl-CoA:succinate CoA transferase in *Propionibacterium freudenreichii* subsp. *shermanii* resulted in diverted carbon fluxes toward PA and higher PA content (Wang et al., 2015). The enzyme transfers the CoA group of the propionyl-CoA product to succinate and primes the succinate to facilitate propionate decarboxylation (Haller et al., 2000). Consequently, we attempted to link the central metabolic pathway with propionyl-CoA-based PA production by overexpressing the propionyl-CoA:succinate CoA transferase (YgfH) gene from *E. coli* in EM42:ΔCE. The resulting EM42:ΔCE:*ygfH* strain produced 2.4 g/L of PA under the same culture conditions, which was 7-fold higher than that produced by EM42:ΔCE (Figure 2A). It has been reported that YgfH not only cycles the CoA pool between propionyl-CoA and succinate-CoA but also influences the carbon flow toward PA production (Wang et al., 2015). The EM42:ΔCE:*ygfH* strain did not consume the LA completely, and 1.8 g/L of residual LA was still available after 48 h of incubation. However, this carbon flow was not equivalent to EM42:ΔCE:*yciA* and hence resulted in a lower PA production (25%) and molar yield (11%) (Figure 2A). These findings thus revealed that the expression of YgfH could improve the PA production in the EM42:ΔCE strain. Furthermore, a plasmid was constructed with the transcriptional fusion of *yciA* and *ygfH* under the same LA-inducible (LvaR/P_*lvaA*_) promoter system and transformed into EM42:ΔCE to obtain the EM42:ΔCE:*yciA*:*ygfH* strain. Our results showed that the co-expression of exogenous YciA and YgfH remarkably improved the PA production by up to 4.6 g/L (Figure 2A). Moreover, as compared to EM42:ΔCE:*yciA* and EM42:ΔCE:*ygfH*, the co-expression of both genes in the EM42:ΔCE increased the PA production by 30 and 48%, respectively. The EM42:ΔCE:*yciA*:*ygfH* also improved the molar yield to 72%, which indicates the maximum carbon flow toward PA production. However, the fact that the remaining 28% of LA was not converted into PA needs to be further investigated. As discussed in the previous findings that the EM42:ΔCE also consumed 4.5 g/L of LA; however, only 0.3 g/L of PA was produced. These findings, therefore, confirmed that the consumed LA is metabolized by unknown enzymes or pathways in *P. putida* other than those involved in the PA production. These unknown enzymes must be revealed in future investigations to further facilitate the improved PA production in *P. putida* by accumulating more propionyl-CoA.

### Selection of glycerol as co-substrate

The results of the previous experiments indicate a low carbon flow availability for the bacterial biomass in the case of EM42:ΔCE:*yciA*:*ygfH*-mediated production of PA. This finding was further confirmed by the reduced growth of EM42:ΔCE:*yciA*:*ygfH* as compared to that of the EM42:ΔCE:*yciA*, EM42:ΔCE:*ygfH,* and EM42:ΔCE strains (Figure 2B). Although the EM42:ΔCE:*yciA*:*ygfH* strain produced a higher PA titer and molar yield, this growth defect must be addressed before large-scale fermentation. To resolve this issue, glycerol was used as a co-substrate because it is known to provide high reducing power and has no catabolic repression with LA (Nikel et al., 2014). Accordingly, 5 g/L glycerol was initially added separately and then in combination with LA (10 g/L), and the growth, substrate(s) consumption, and PA production were estimated in all the four strains, including EM42:ΔCE, EM42:ΔCE:*yciA*:*ygfH*, EM42:ΔCE:*ygfH*, and EM42:ΔCE:*yciA*:*ygfH* (Figures 2C,F). Subsequent analysis revealed that the media containing glycerol alone initially showed no growth differences among the four strains (Figure 2D). However, the expression of YciA and/or YgfH showed no significant difference in PA production, possibly because glycerol could not provide sufficient propionyl-CoA as a precursor for PA production (Figure 2C). In contrast, propionyl-CoA can be generated from the degradation of the amino acids L-methionine, L-isoleucine, and L-valine and as an end-product of the β-oxidation of uneven fatty acids (Thompson et al., 2020). In this study, the nutrient-rich TB medium was used, which provided these amino acids as a source of propionyl-CoA. Hence, while using glycerol as a substrate, 0.6–0.7 g/L PA was produced from all four strains despite the expression of both enzymes. Subsequently, both substrate (LA) and co-substrate (glycerol) were added to the medium and cultivated for 48 h. Our results revealed that the addition of glycerol showed no growth defects that in this case, and all the four strains displayed a remarkably similar growth profile (Figure 2F). Furthermore, the PA production was enhanced in all four strains, but the maximum PA production was achieved by EM42:ΔCE:*yciA*:*ygfH* strain at 6.5 g/L (Figure 2E). These results validate the selection of glycerol as a co-substrate, as it is required to eliminate growth defects and improve PA production. Moreover, our results also revealed that the EM42:ΔCE:*yciA*:*ygfH* strain completely utilized glycerol and LA after 48 h of incubation. These findings again confirmed that the propionyl-CoA generated from the media source also contributed to the PA, as in the previous experiments, PA was produced although at a lower capacity when glycerol alone was used as a carbon source (Figures 2C,E).

### Production of propionic acid in fed-batch bioreactor cultivation

**FIGURE 3:**
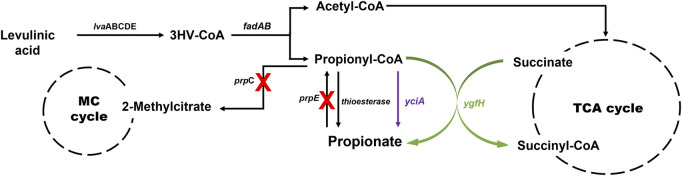
Metabolic pathway involved in PA production from LA in *P. putida*. The *yciA* (encoding thioesterase from *H. influenzae*) and *ygfH* (encoding propionyl-CoA: succinate CoA transferase from *E. coli*) were overexpressed, and the *prpC* and *prpE* genes encoding methylcitrate synthase and propionyl-CoA synthase, respectively, were deleted. MC cycle, methylcitrate cycle; TCA cycle, tricarboxylic acid cycle; *lvaABCDE*, *lva* operon of *P. putida* involved in LA degradation.

**FIGURE 4:**
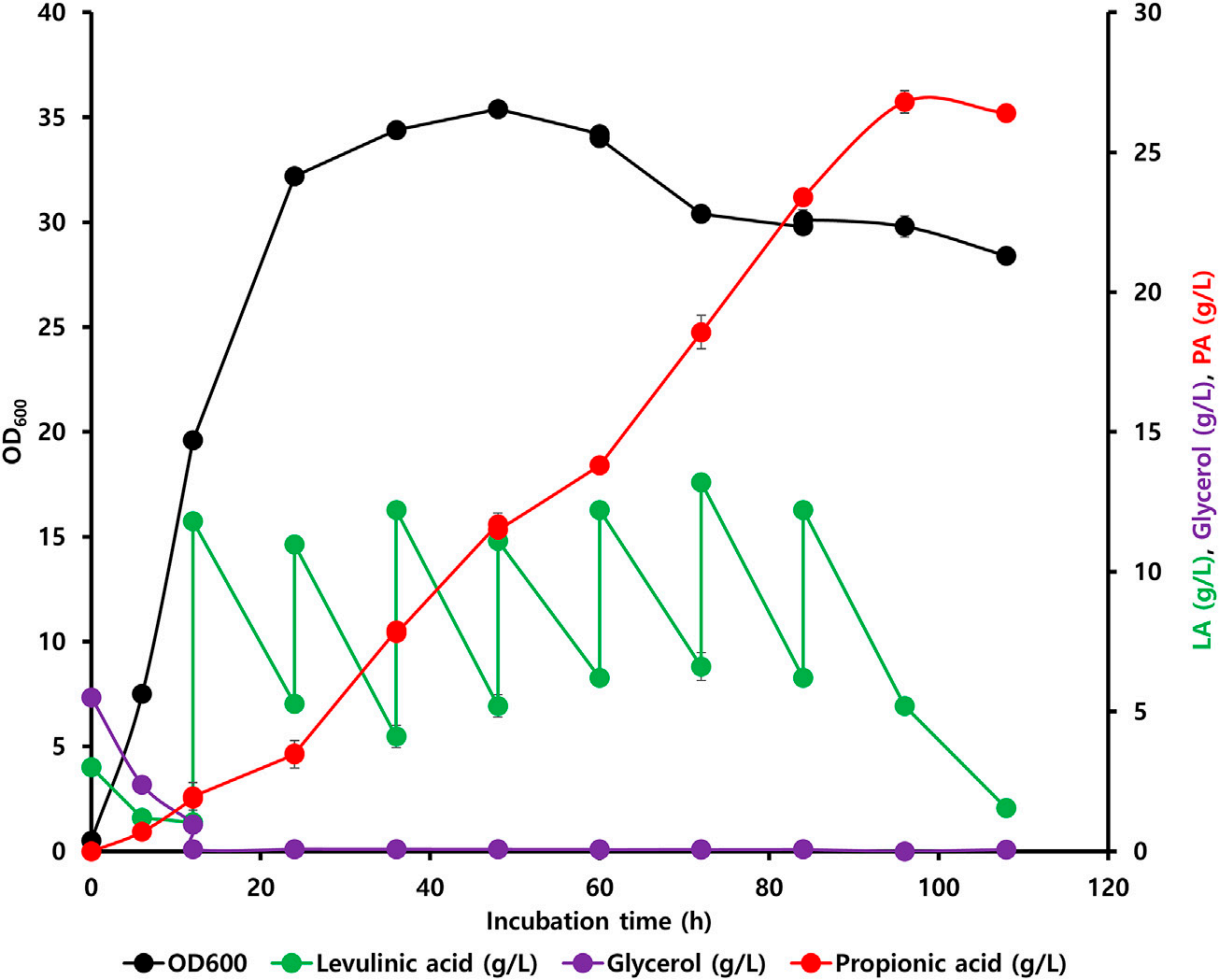
Fed-batch fermentation led by the engineered EM42:ΔCE:*yciA*:*ygfH* strain exhibiting an LA-based PA synthesis pathway.

Initially, the accumulation of propionyl-CoA in the EM42 strain was enhanced by deleting the PrpC gene responsible for its assimilation through the methylcitrate cycle. Likewise, the interconversion of PA to propionyl-CoA was blocked by the deletion of the *prpE*. Subsequently, the final strain, EM42:ΔCE:*yciA*:*ygfH,* was obtained after co-expressing YciA and YgfH, which are responsible for the PA production and diverting more carbon flow toward PA (Figure 3). The fed-batch fermentation was then carried out in a 5-L bioreactor using the EM42:ΔCE:*yciA*:*ygfH* strain to validate the PA production on a large scale. The biomass cultivation was initiated by adding 5 g/L glycerol and 3 g/L LA as inducers. Our growth curve assessment revealed that the OD_600_ reached approximately 20 after cultivation for 12 h, followed by the addition of 10 g/L of LA to initiate the PA production (Figure 4). Post-continuous LA supply as a carbon source, the maximum OD_600_ of the culture reached up to 35.4 after 48 h of incubation. Subsequently, the growth stopped, but the PA production continued. Further analysis revealed that the maximum PA titer of 26.8 g/L with a molar yield of 83% and productivity of 0.3 g/L/h was obtained after consuming 49.56 g of LA as a carbon source (Figure 4). By-products, such as succinate and acetate, could not be detected during the entire bioprocess. These findings are thus indicative of high molar yield at the flask scale as well as large-scale fermentation and, therefore, significant from an economic point of view. Hence, the selection of low-cost substrates such as LA and a high molar yield can enhance the profit margin for the industrial production of PA (Ahmadi et al., 2017). However, further optimization in large-scale fermentation needs to be conducted to enhance the PA titer to be comparable with *Propionibacterium* candidates. In this study, an enriched TB medium was used to produce PA. In future, optimization of growth components is required to render the medium cost to expand not only the sustainability of complete bioprocess but also further improve the PA productivity and titer. Moreover, compared to previous report where L-threonine was used as a substrate, the higher titer of 62 g/L and productivity of 1.07 g/L/h was achieved by the fed-batch biotransformation coupled process (Mu et al., 2021). Thus, while high-density culture can increase productivity and titer, biotransformation on an industrial scale is a difficult process.

## Conclusion

In conclusion, to the best of our knowledge, the present study is the first to demonstrate the application of LA, a new renewable low-cost substrate, in the production of PA using metabolically engineered *P. putida*. In the reported bioproduction system, the accumulation of propionyl-CoA as a precursor for PA was confirmed by deleting alternative pathways. Moreover, the recombinant strains were developed using an LA-inducible promoter system, which eliminated the use of costly inducer chemicals and the extra metabolic burden of the engineered strain. After the selection and expression of the required thioesterase gene in the engineered strains, it was observed that the PA production level was significantly increased as compared to that achieved by the parental strains. Likewise, the molar yield and carbon flow distribution were enhanced by the co-expression of YgfH. Additionally, glycerol as a co-substrate was selected to abolish the growth defect and enhance PA titer and productivity. Furthermore, the efficiency of the engineered strain for PA production was validated by large-scale fed-batch fermentation. Our findings thus suggest that the engineered *P. putida* strain combined with a developed process can be used as a cost-effective and sustainable bioprocess for PA production.

## Data Availability

The original contributions presented in the study are included in the article/Supplementary Material, further inquiries can be directed to the corresponding author.
