# Supplementary material for: Bioproduction of propionic acid using levulinic acid by engineered Pseudomonas putida
Source: Front Bioeng Biotechnol. 2022 Aug 10;10:939248. doi: 10.3389/fbioe.2022.939248 (PMC9399607; doi:10.3389/fbioe.2022.939248)
Supplement: Supplementary file 1 [file Table1.DOCX]

Supplementary Material

**Supplementary Table 1** Primers used in this study

| **Primers** | **Primer sequence (**5ʹ **to** 3ʹ**)** | **Purpose** |
| --- | --- | --- |
| HiYciA_F | ctttataaggaggaaaaacatatgtctgccaattttactg | Primers used to amplify and clone *yciA* gene (HI0827) from *H. influenzae* |
| HiYciA_R | agtccaagctcagctaattaagcttacaagggttgttctgaaattaag |  |
| HiYciA_RBS_R | atgtatatctccttcttaaagttacaagggttgttctgaaattaag |  |
| YgfH_F | ctttataaggaggaaaaacatatggaaactcagtggacaag | Primers used to amplify and clone *ygfH* gene from *E. coli* |
| YgfH_RBS_F | ctttaagaaggagatatacatatggaaactcagtggacaag |  |
| YgfH_R | agtccaagctcagctaattaagcttaacccagcatcgagcc |  |
| UP_2335_F | cgaaaagtgccacctgacgt*c*gcttcgtgatcatggcgc | Primers used to delete *prpC* (PP2335) gene from EM42 |
| UP_2335_R | gctattcagcggccatggtgtttctcctttc |  |
| DS_2335_F | caccatggccgctgaatagctatagagaggcaaccc |  |
| DS_2335_R | caaataaattttttatgatttc*tcgag*caccgggttgacctgggc |  |
| PP_2335_F | gcgctgccgccatccata |  |
| PP_2335_R | gcccggctggatcacgt |  |
| UP_2351_F | cgaaaagtgccacctgacgtcatcatcgggttgcccag | Primers used to delete *prpE* (PP2351) gene from EM42 |
| UP_2351_R | aaaccatcaactcatgtcgttagaaacccg |  |
| DS_2351_F | acgacatgagttgatggtttgacctcgatggccctttcgc |  |
| DS_2351_R | caaataaattttttatgatttc*tcga*gcgcagcgccaggcgctg |  |
| PP_2351_F | ccatctcgaccttgcgca |  |
| PP_2351_R | cgtggcgtgtacctgct |  |
| pQSAK F | ctgggtgagcaaaaacagga | For sequencing of pQSAK_constructs |
| pQSAK R | ctcaagcgaaaggaaacaat |  |
| pPROBE-Seq-F | caggaattggggatcggaag | For sequencing of pPROBE_constructs |
| pPROBE-Seq-R | cgccaagctagcttggattctc |  |
